# Supplementary material for: Hepatic MIR20B promotes nonalcoholic fatty liver disease by suppressing PPARA
Source: eLife. 2021 Dec 29;10:e70472. doi: 10.7554/eLife.70472 (PMC8758141; doi:10.7554/eLife.70472)
Supplement: Source data 1. [file elife-70472-supp1.zip › Source data- Western blot.pptx]

## Slide 1
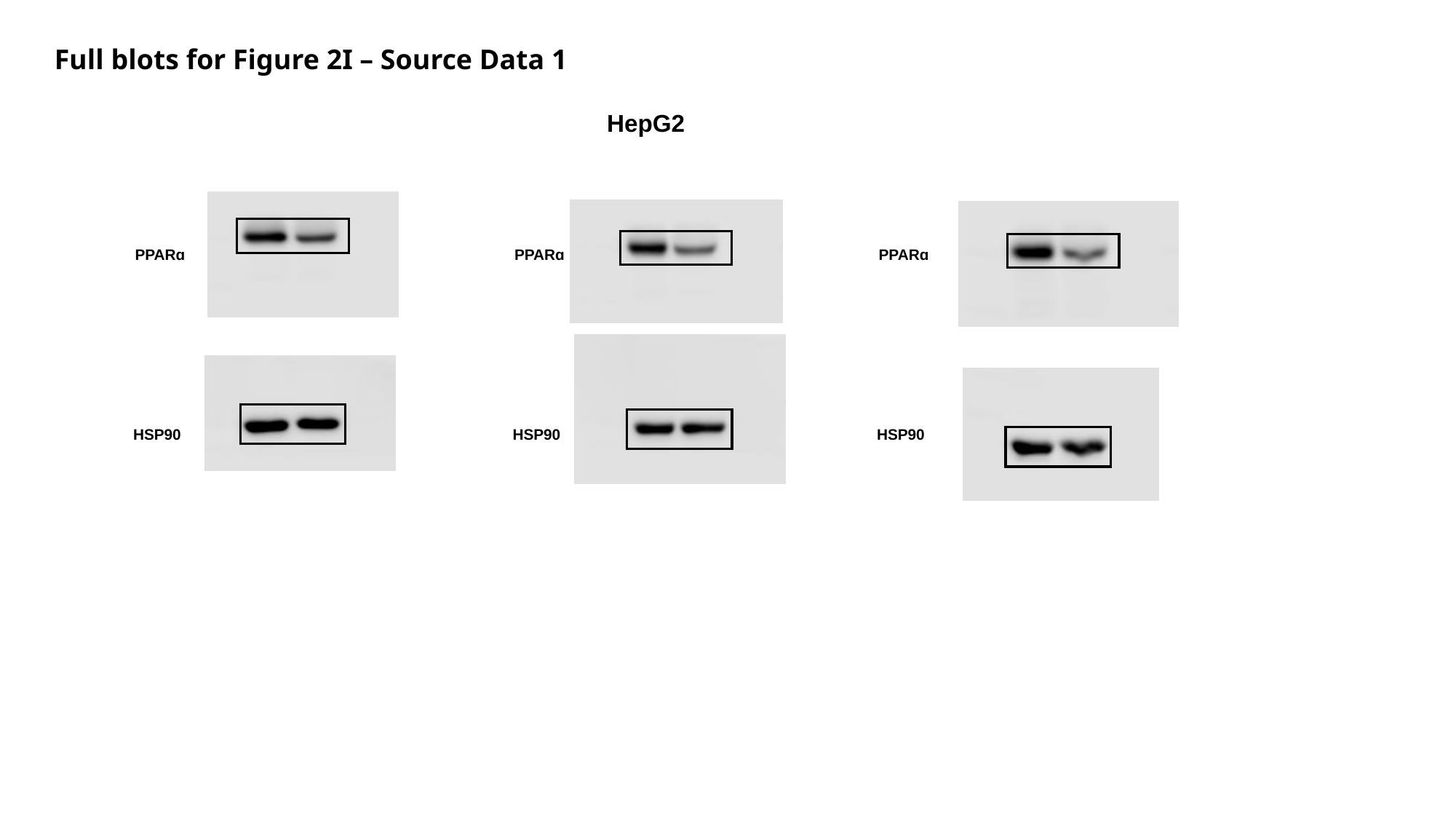

Full blots for Figure 2I – Source Data 1
HepG2
PPARɑ
PPARɑ
PPARɑ
HSP90
HSP90
HSP90

## Slide 2
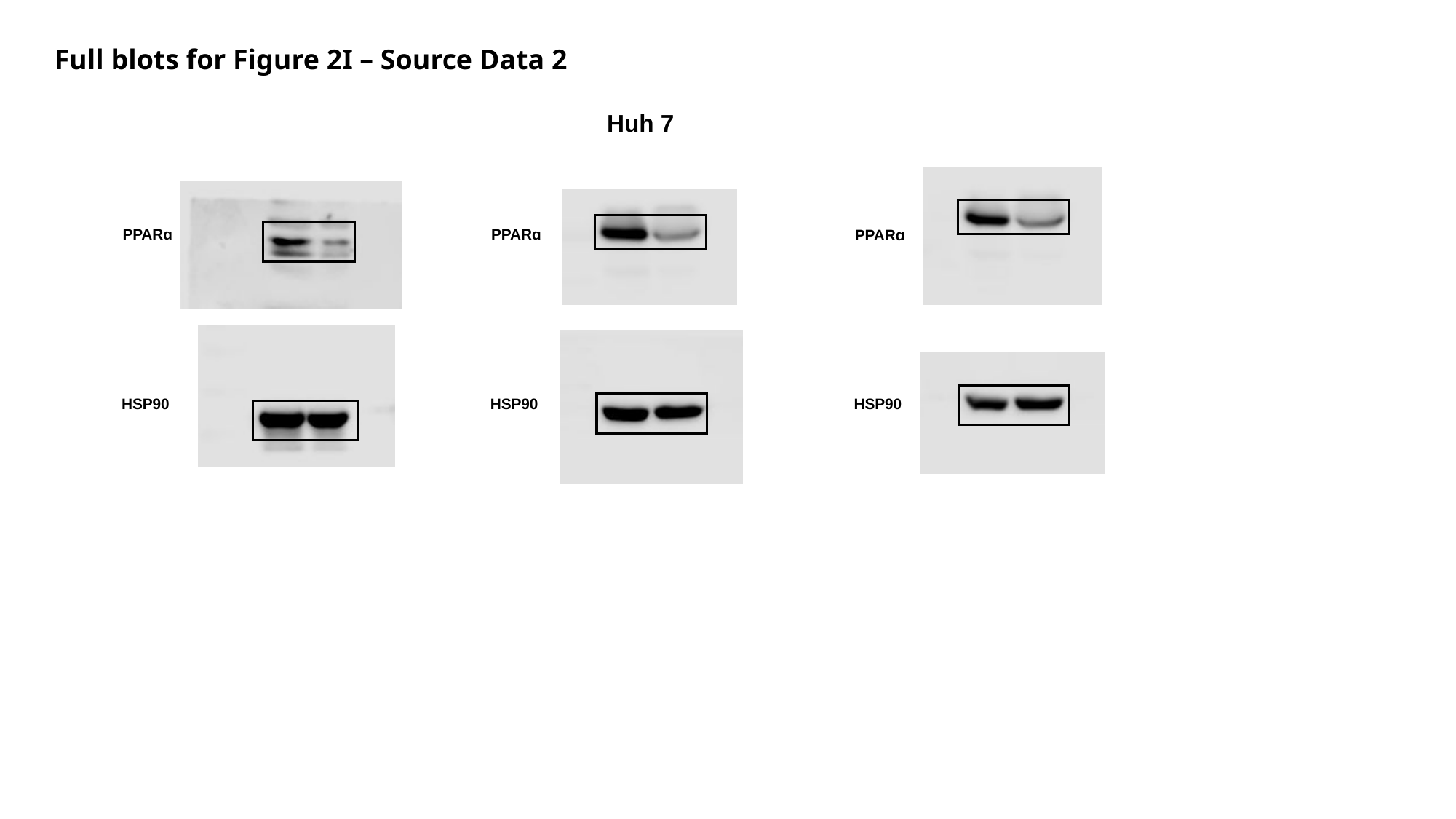

Full blots for Figure 2I – Source Data 2
Huh 7
PPARɑ
PPARɑ
PPARɑ
HSP90
HSP90
HSP90

## Slide 3
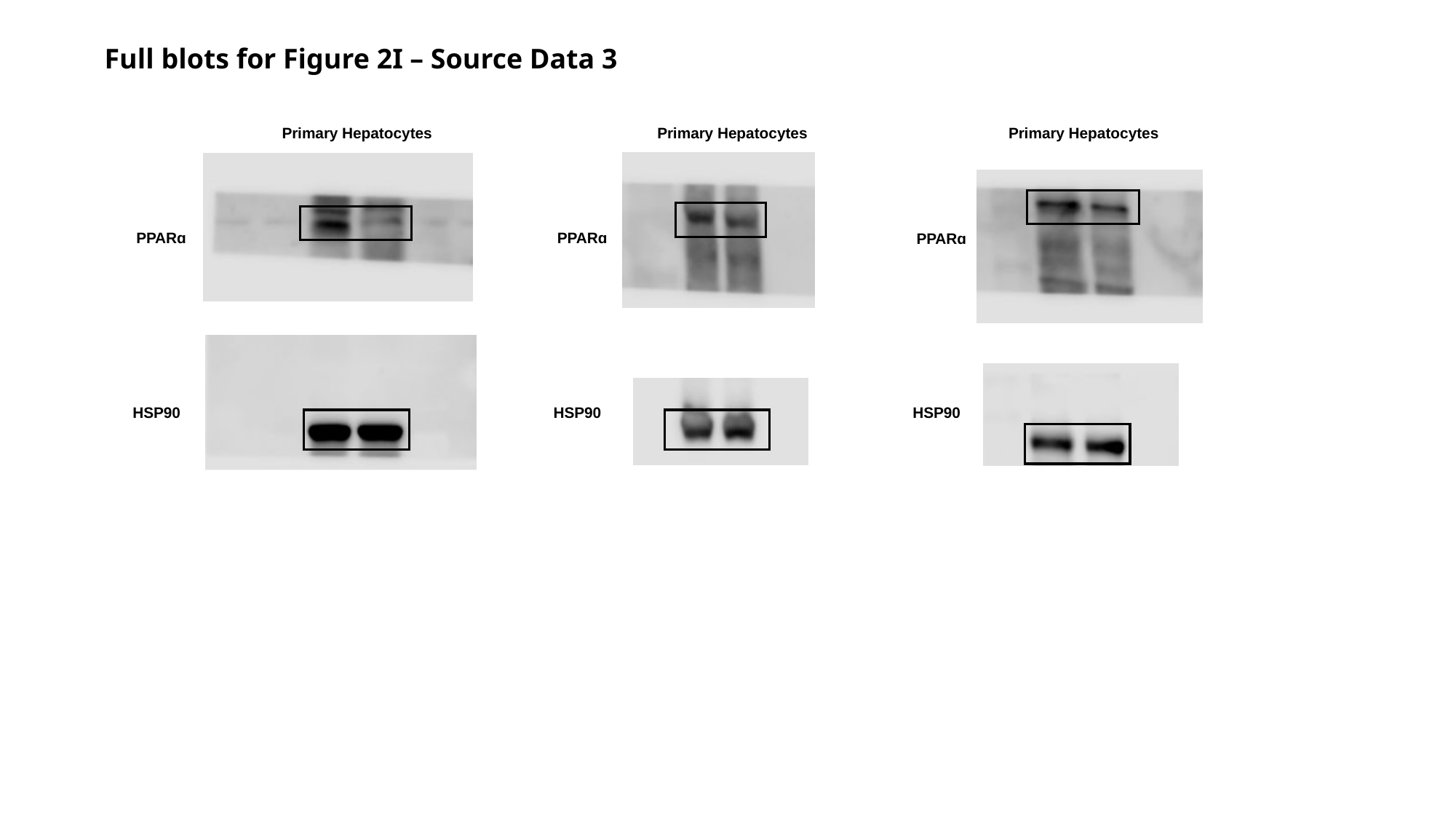

Full blots for Figure 2I – Source Data 3
Primary Hepatocytes
Primary Hepatocytes
Primary Hepatocytes
PPARɑ
PPARɑ
PPARɑ
HSP90
HSP90
HSP90

## Slide 4
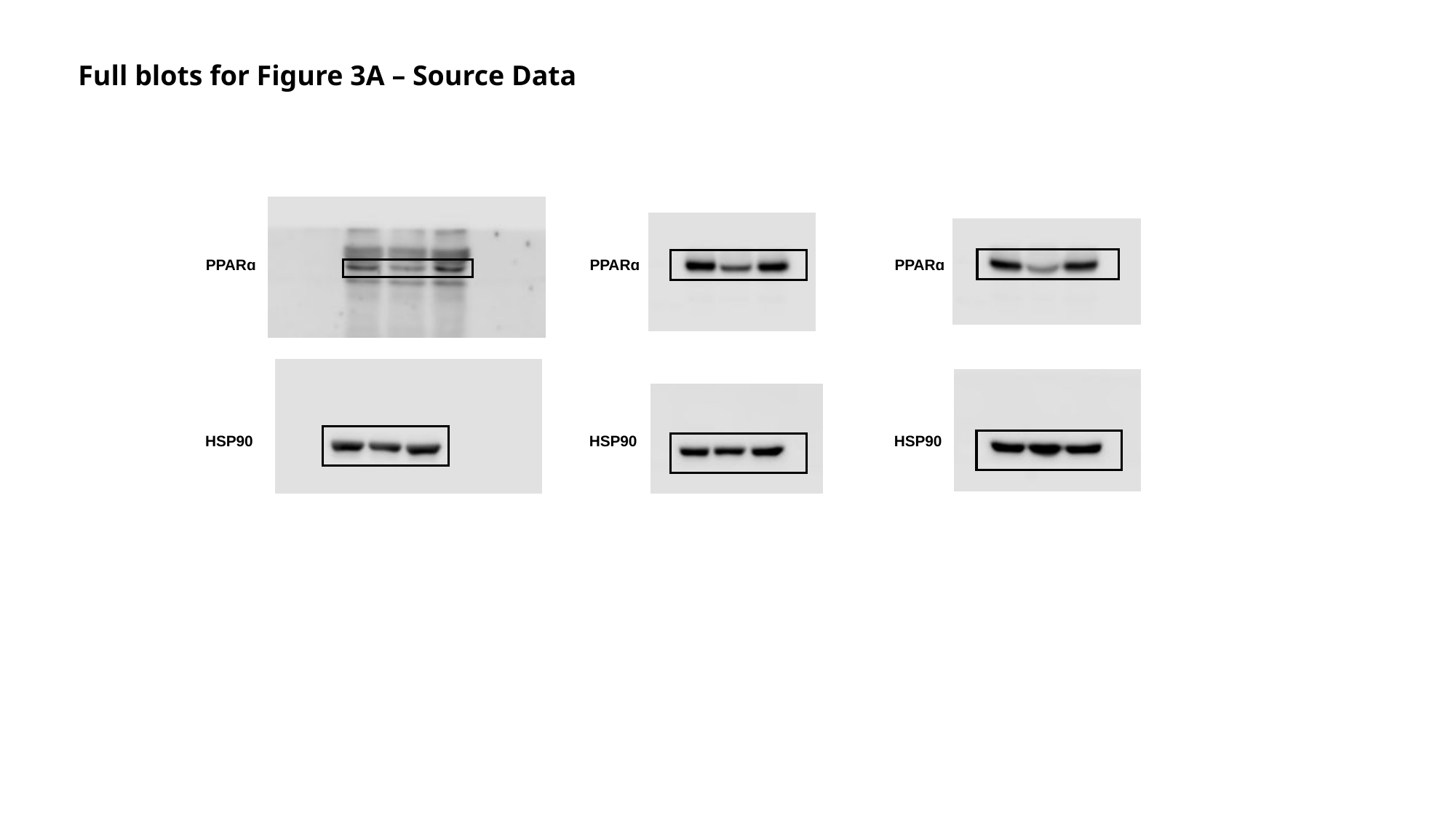

Full blots for Figure 3A – Source Data
PPARɑ
PPARɑ
PPARɑ
HSP90
HSP90
HSP90

## Slide 5
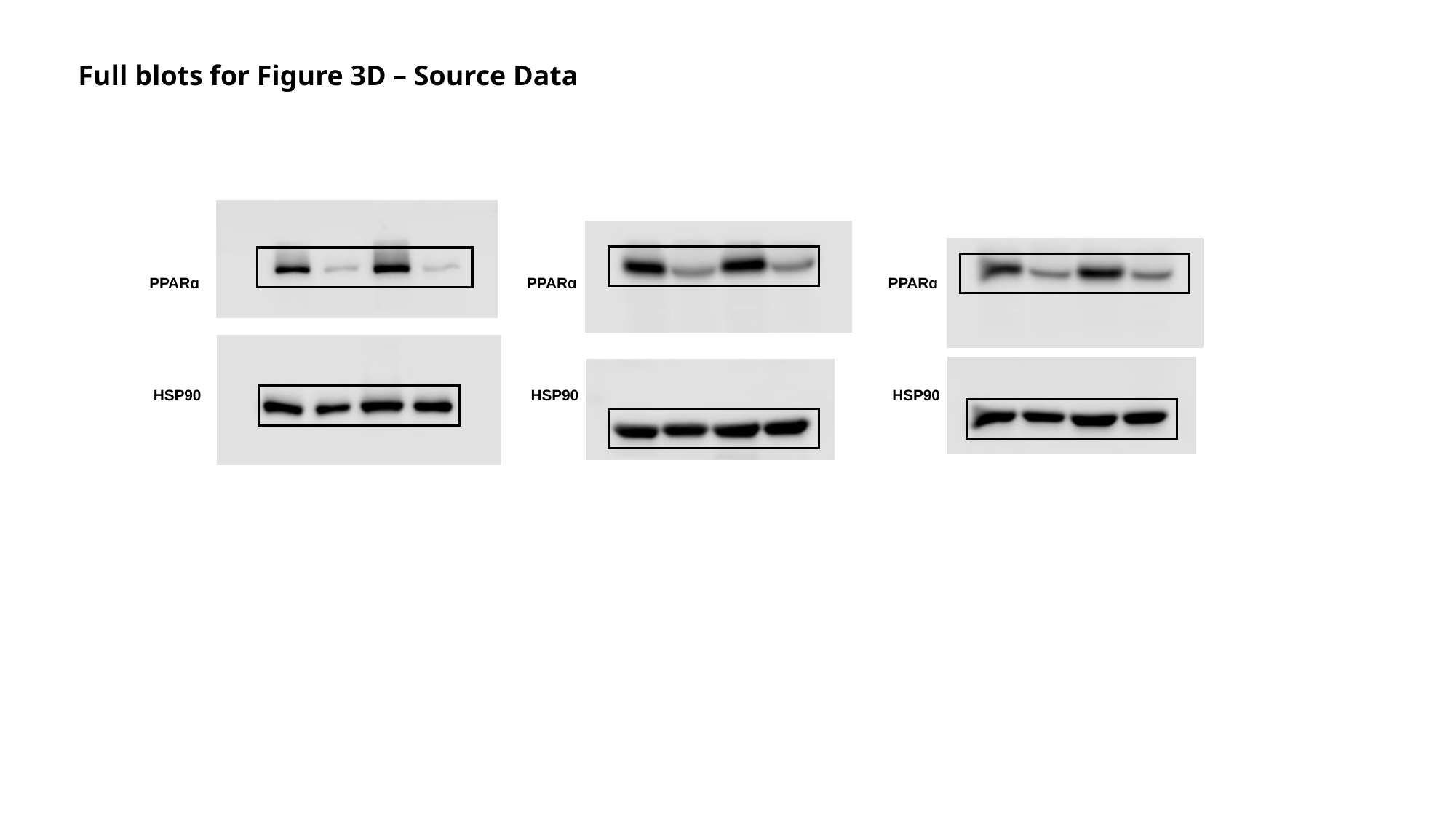

Full blots for Figure 3D – Source Data
PPARɑ
PPARɑ
PPARɑ
HSP90
HSP90
HSP90

## Slide 6
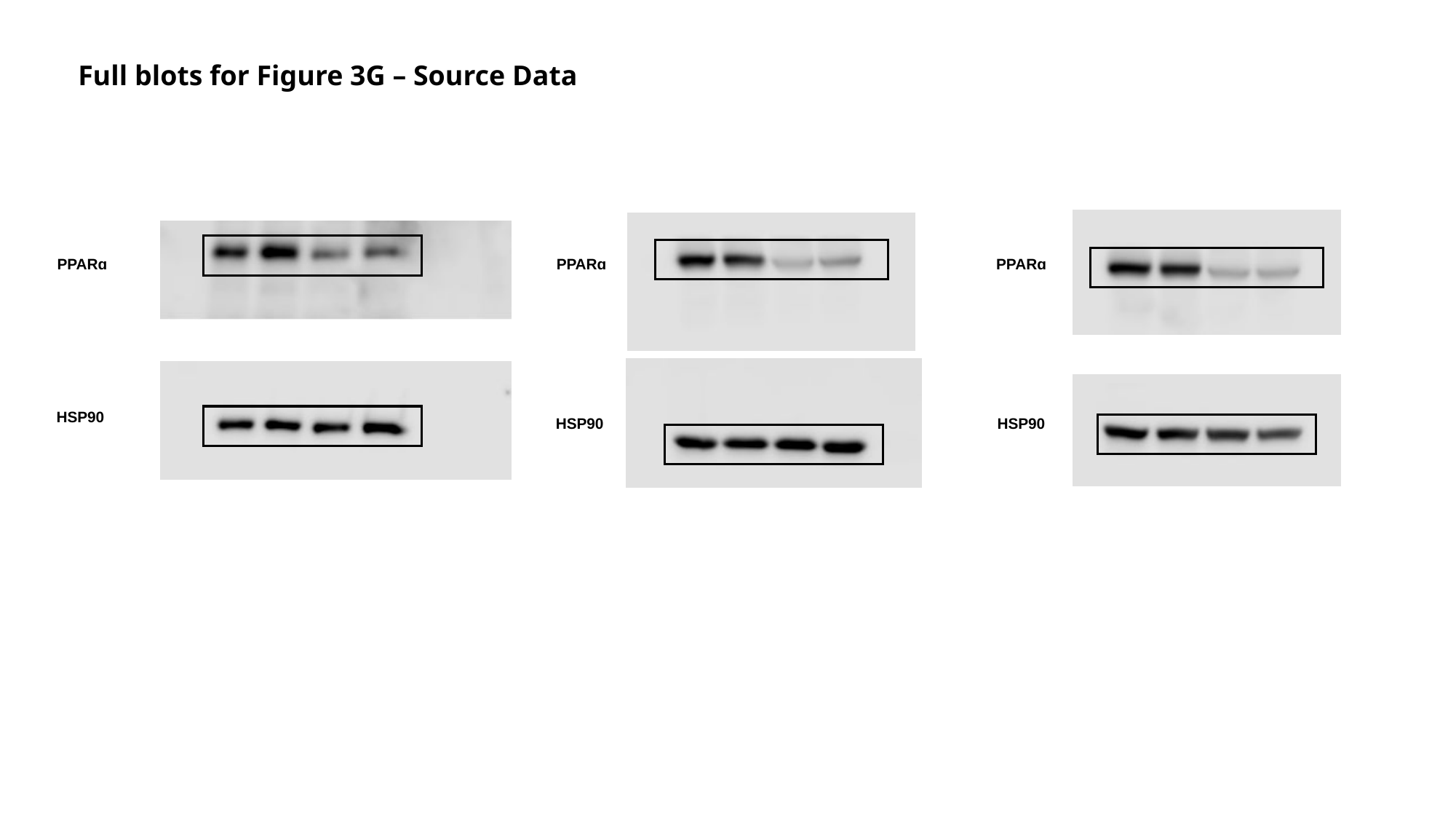

Full blots for Figure 3G – Source Data
PPARɑ
PPARɑ
PPARɑ
HSP90
HSP90
HSP90

## Slide 7
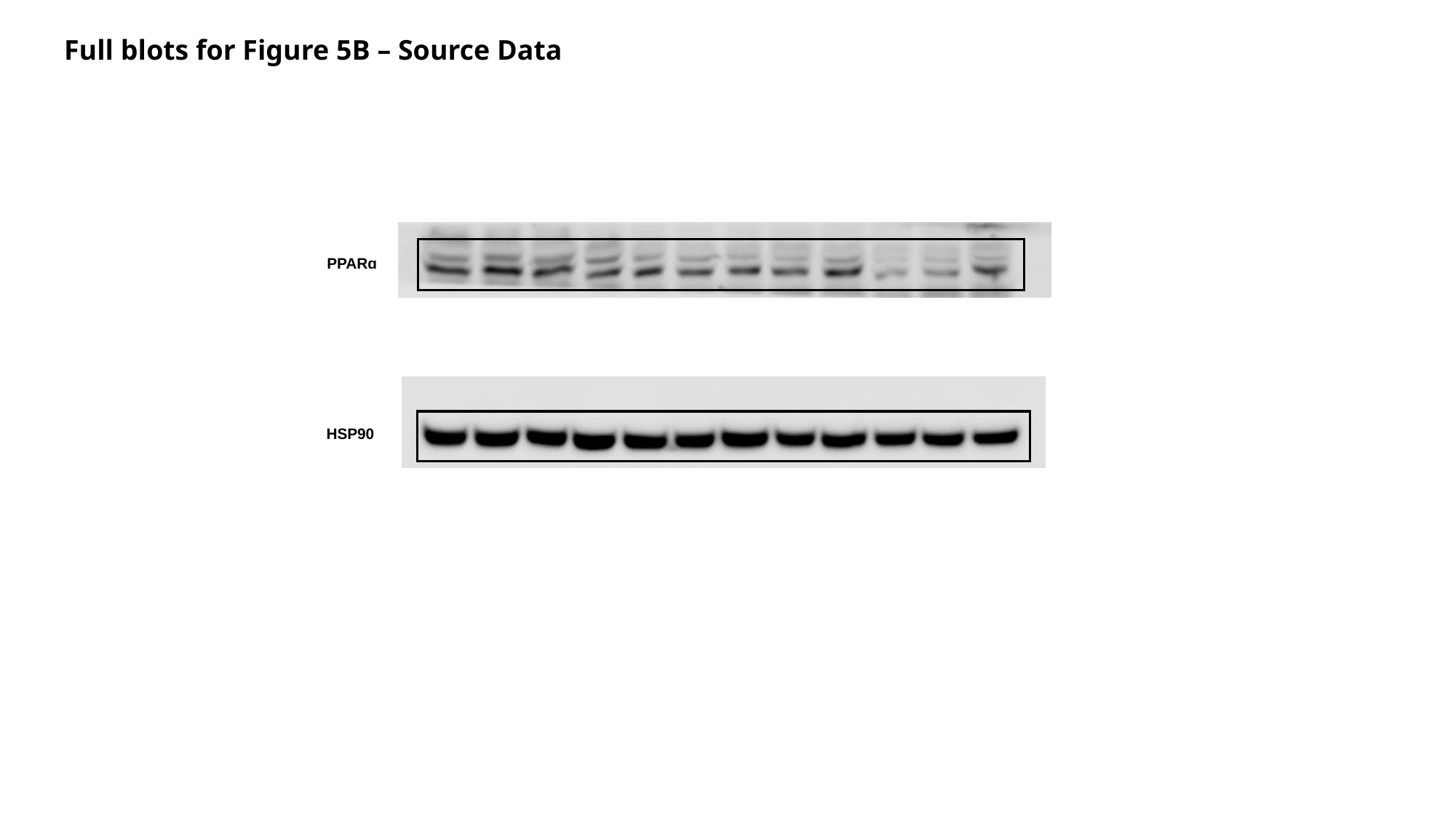

Full blots for Figure 5B – Source Data
PPARɑ
HSP90

## Slide 8
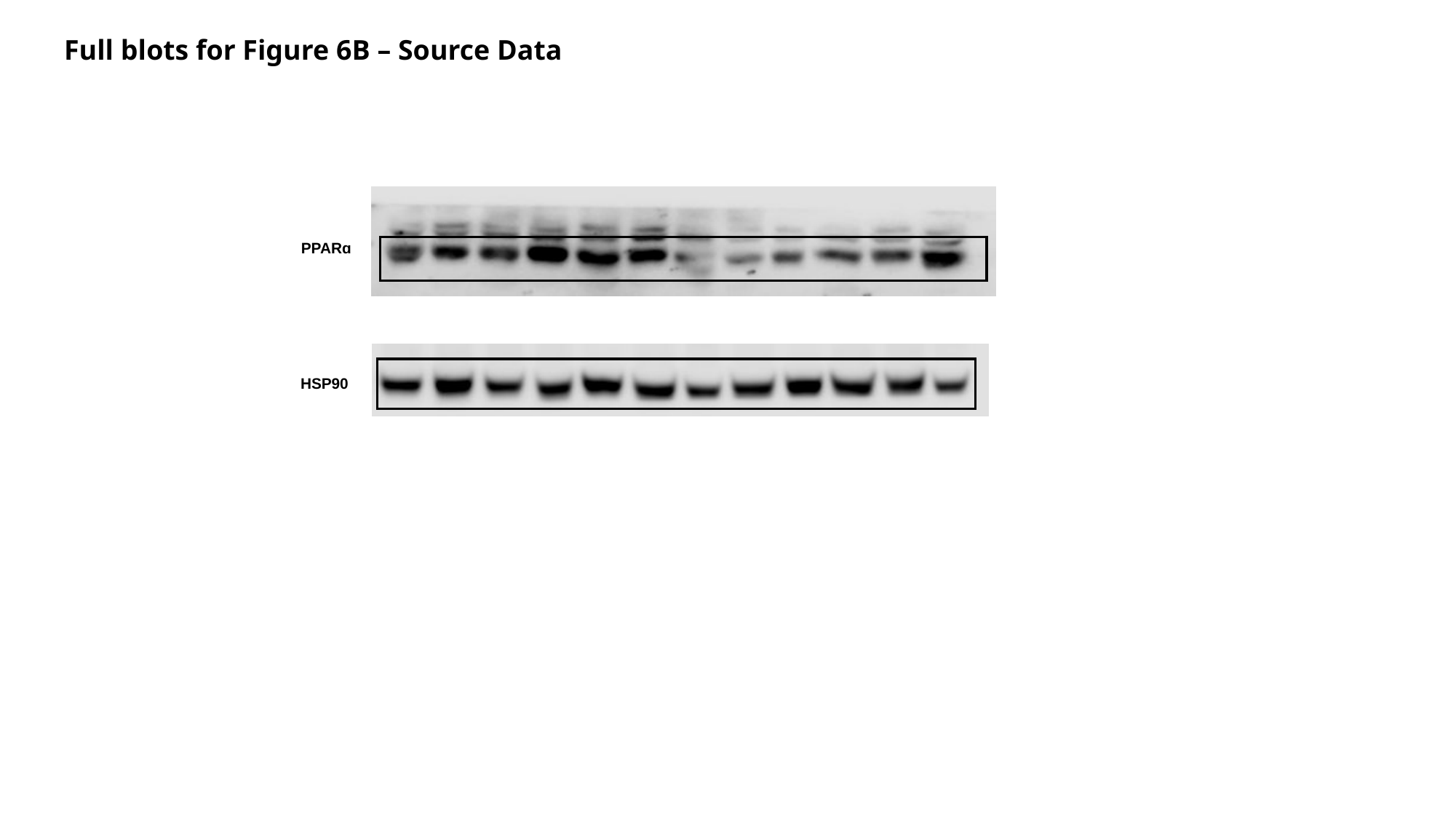

Full blots for Figure 6B – Source Data
PPARɑ
HSP90

## Slide 9
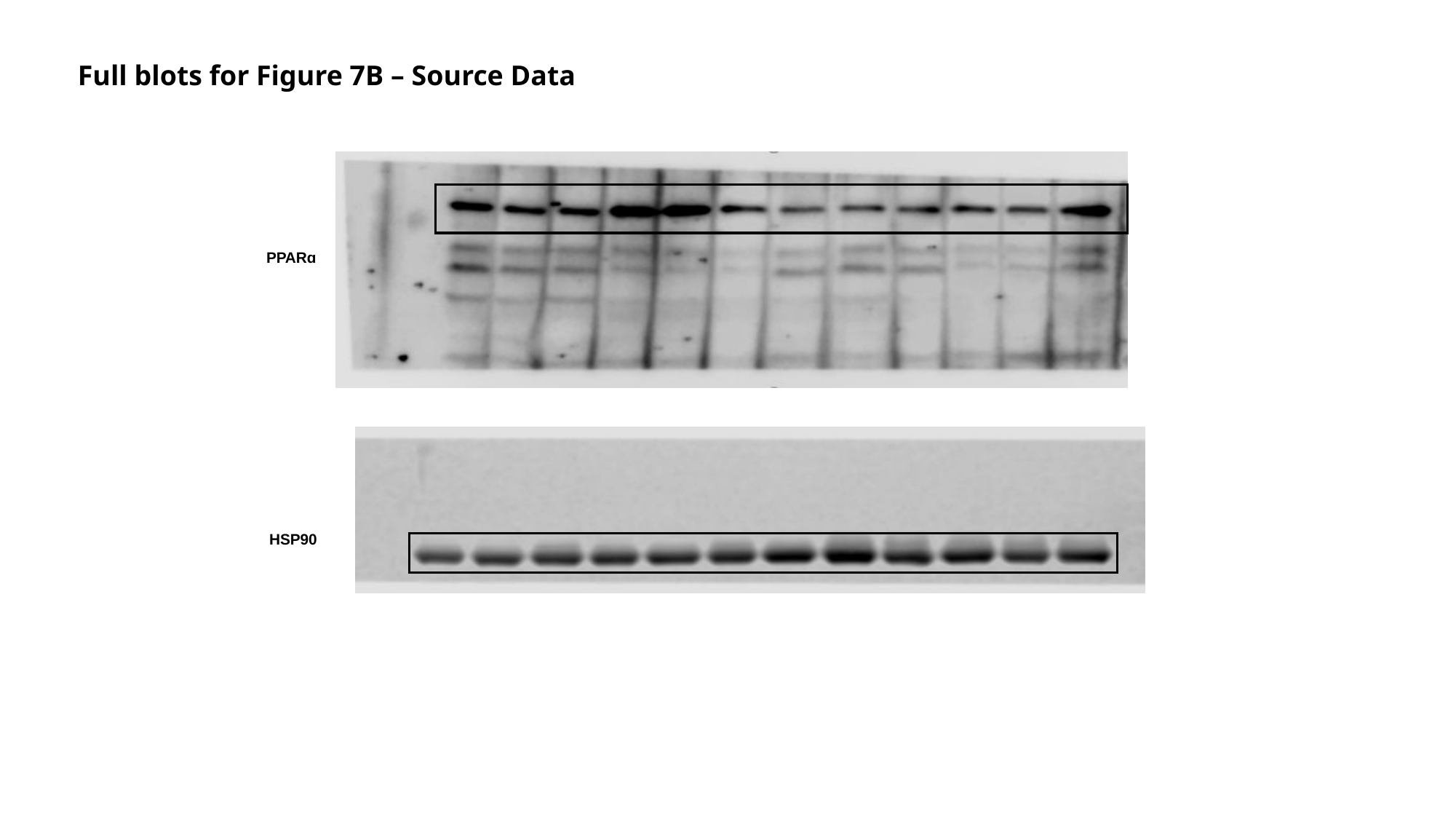

Full blots for Figure 7B – Source Data
PPARɑ
HSP90

## Slide 10
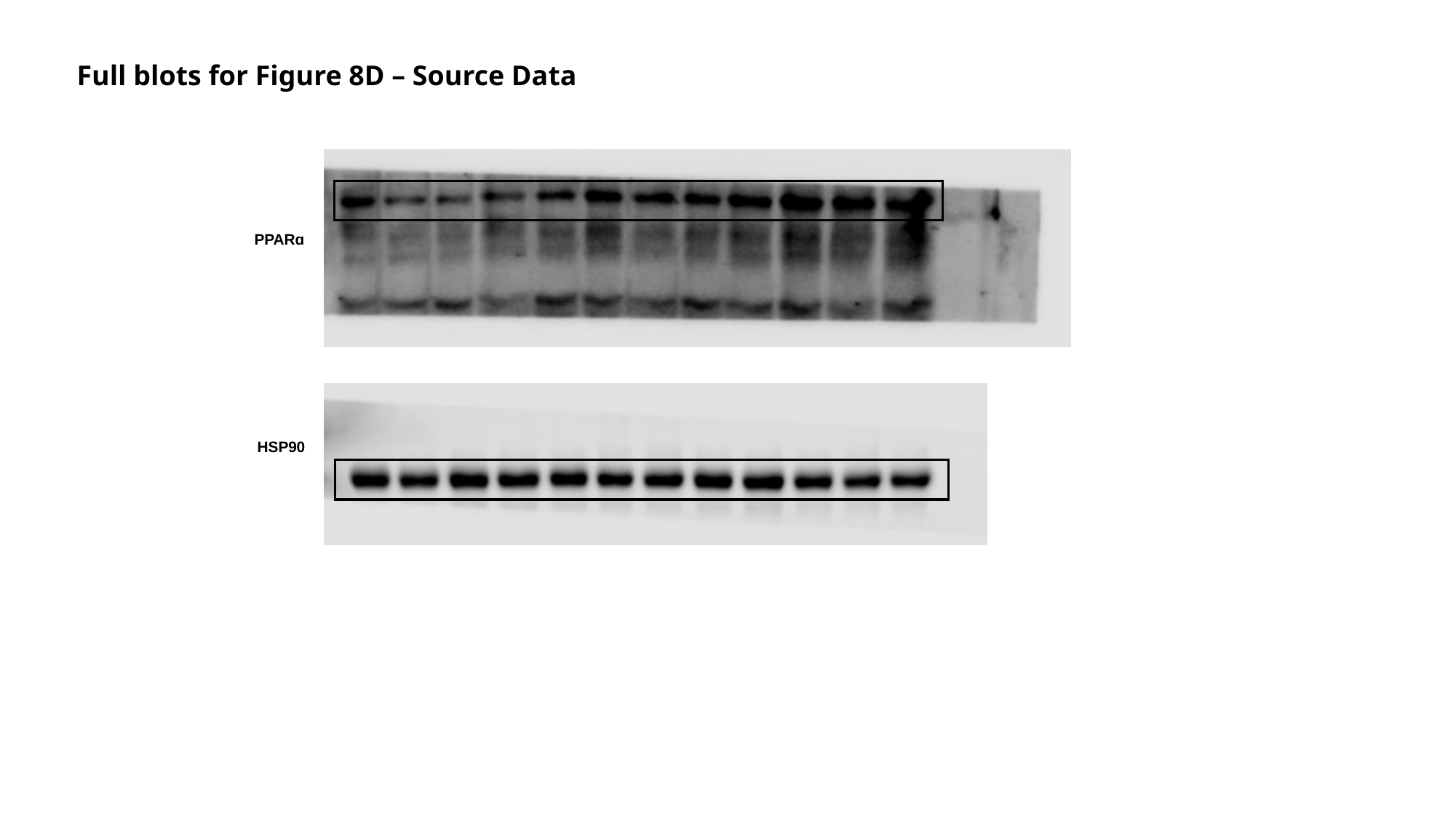

Full blots for Figure 8D – Source Data
PPARɑ
HSP90

## Slide 11
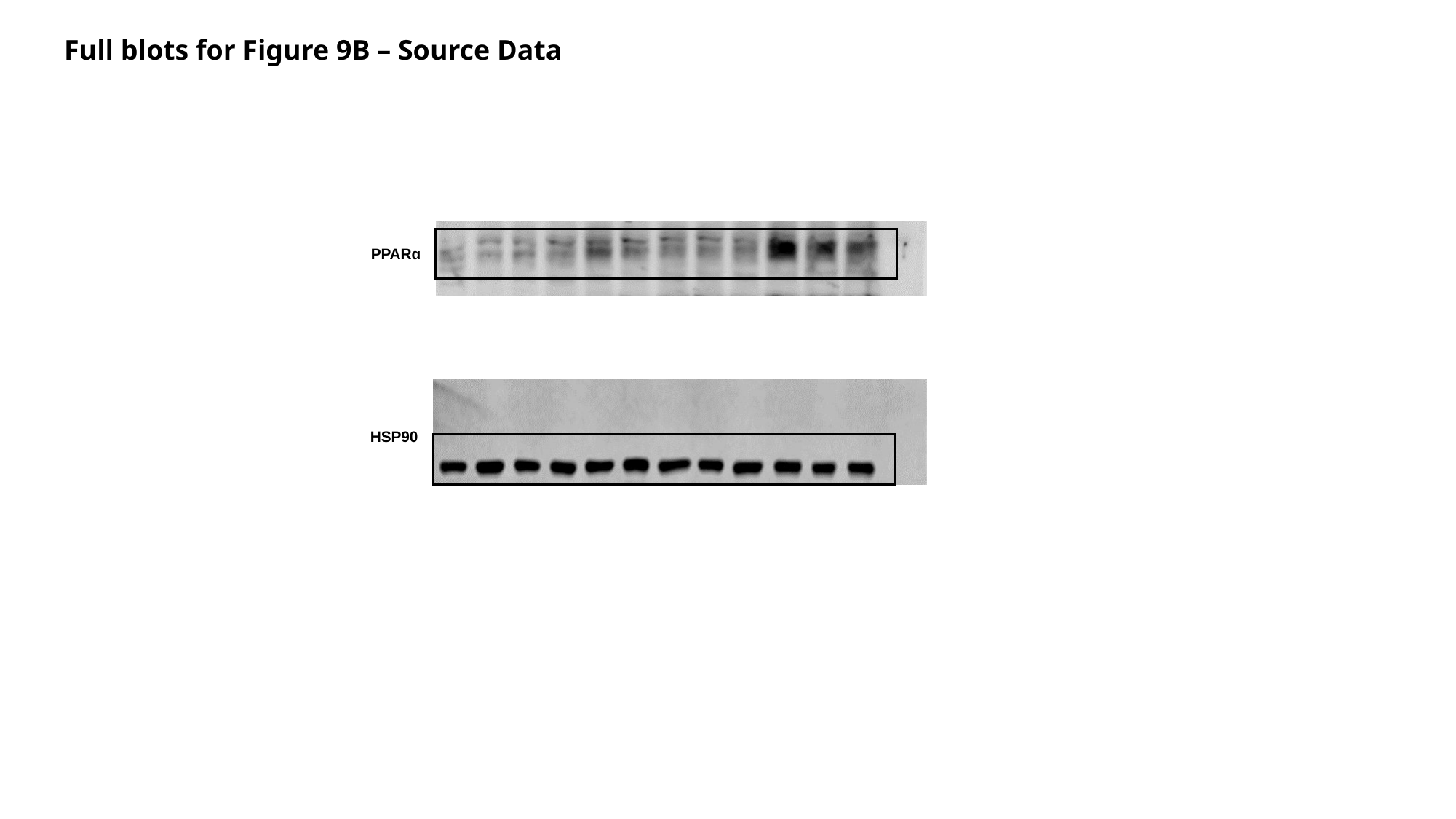

Full blots for Figure 9B – Source Data
PPARɑ
HSP90
