# Supplementary material for: Hepatic MIR20B promotes nonalcoholic fatty liver disease by suppressing PPARA
Source: eLife. 2021 Dec 29;10:e70472. doi: 10.7554/eLife.70472 (PMC8758141; doi:10.7554/eLife.70472)
Supplement: Transparent reporting form [file elife-70472-transrepform1.docx]

***eLife’s* transparent reporting form**

We encourage authors to provide detailed information *within their submission* to facilitate the interpretation and replication of experiments. Authors can upload supporting documentation to indicate the use of appropriate reporting guidelines for health-related research (see [EQUATOR Network](http://www.equator-network.org/%20)), life science research (see the [BioSharing Information Resource](https://biosharing.org/)), or the [ARRIVE guidelines](http://www.plosbiology.org/article/info:doi/10.1371/journal.pbio.1000412) for reporting work involving animal research. Where applicable, authors should refer to any relevant reporting standards documents in this form.

If you have any questions, please consult our Journal Policies and/or contact us: [editorial@elifesciences.org](mailto:editorial@elifesciences.org).

**Sample-size estimation**

- You should state whether an appropriate sample size was computed when the study was being designed
- You should state the statistical method of sample size computation and any required assumptions
- If no explicit power analysis was used, you should describe how you decided what sample (replicate) size (number) to use

Please outline where this information can be found within the submission (e.g., sections or figure legends), or explain why this information doesn’t apply to your submission:

All details related to sample size are detailed in the materials and methods and figure legends. In addition, we include a table (Table 1) which indicates the number of individuals (patients liver sample) used for each experiment. Moreover, a minimum of 3 technical replicates was always used for each cell-based or *in vivo* research. We used Student’s t-test with each experiment.

**Replicates**

- You should report how often each experiment was performed
- You should include a definition of biological versus technical replication
- The data obtained should be provided and sufficient information should be provided to indicate the number of independent biological and/or technical replicates
- If you encountered any outliers, you should describe how these were handled
- Criteria for exclusion/inclusion of data should be clearly stated
- High-throughput sequence data should be uploaded before submission, with a private link for reviewers provided (these are available from both GEO and ArrayExpress)

Please outline where this information can be found within the submission (e.g., sections or figure legends), or explain why this information doesn’t apply to your submission:

Experiment frequency and the number of mice and cells are indicated in figure legends for each relevant experiment and/or in the materials and methods section. We define independent biological replicates as experiments performed with sample collected from different cell cultures or mice on separate days. Most experiments were performed 3 times with different numbers of biological replicates (cells) of n=3 as indicated in each figure. In addition, for transcriptional analysis by RT-qPCR, we used technical replicates, which in this case means analysis of the same cDNA samples in the multiple wells of a qPCR plate. RNA-sequencing data from this study have been deposited in the Gene Expression Omnibus (GEO) under the accession number GSE168484.

**Statistical reporting**

- Statistical analysis methods should be described and justified
- Raw data should be presented in figures whenever informative to do so (typically when N per group is less than 10)
- For each experiment, you should identify the statistical tests used, exact values of N, definitions of center, methods of multiple test correction, and dispersion and precision measures (e.g., mean, median, SD, SEM, confidence intervals; and, for the major substantive results, a measure of effect size (e.g., Pearson's r, Cohen's d)
- Report exact p-values wherever possible alongside the summary statistics and 95% confidence intervals. These should be reported for all key questions and not only when the p-value is less than 0.05.

Please outline where this information can be found within the submission (e.g., sections or figure legends), or explain why this information doesn’t apply to your submission:

Statistical analysis is described in each figure legends, as well as the Statistical analysis section of the materials and methods. *P* values are presented within the figures and the figure legends.

All data are represented as mean ± SEM. Statistically significant differences were assessed by the Student’s t-test. Statistical analyses were performed using Microsoft Excel or GraphPad Prism 9. All of the significance are expressed as **P* < 0.05, ***P* < 0.01, ****P* < 0.001, ^#^*P* < 0.05, ^##^*P* < 0.01, ^###^*P* < 0.001, ^$^*P* < 0.05, ^$$^*P* < 0.01, and ^$$$^*P* < 0.001.

(For large datasets, or papers with a very large number of statistical tests, you may upload a single table file with tests, Ns, etc., with reference to sections in the manuscript.)

**Group allocation**

- Indicate how samples were allocated into experimental groups (in the case of clinical studies, please specify allocation to treatment method); if randomization was used, please also state if restricted randomization was applied
- Indicate if masking was used during group allocation, data collection and/or data analysis

Please outline where this information can be found within the submission (e.g., sections or figure legends), or explain why this information doesn’t apply to your submission:

Only healthy mice (each group of pooled mice consisted of 3 - 10 mice, co-housed mice of the same strain, gender and age) or human liver tissue samples (defined as normal individuals, Steatosis and NASH) were considered in our analysis. Therefore, randomization and masking of research was not required.

**Additional data files (“source data”)**

- We encourage you to upload relevant additional data files, such as numerical data that are represented as a graph in a figure, or as a summary table
- Where provided, these should be in the most useful format, and they can be uploaded as “Source data” files linked to a main figure or table
- Include model definition files including the full list of parameters used
- Include code used for data analysis (e.g., R, MatLab)
- Avoid stating that data files are “available upon request”

Please indicate the figures or tables for which source data files have been provided:

Source data files are provided for Figure 1, Figure 1-figure supplement 3, Figure 1-figure supplement 4, Figure 2, Figure 2-figure supplement 2, Figure 2-figure supplement 3, Figure 2-figure supplement 4, Figure 2-figure supplement 5, Figure 3, Figure 4, Figure 4-figure supplement 1, Figure 4-figure supplement 2, Figure 4-figure supplement 3, Figure 4-figure supplement 4, Figure 4-figure supplement 5, Figure 4-figure supplement 6, Figure 5, Figure 5-figure supplement 1, Figure 5-figure supplement 2, Figure 5-figure supplement 3, Figure 5-figure supplement 4, Figure 6, Figure 6-figure supplement 1, Figure 6-figure supplement 2, Figure 6-figure supplement 3, Figure 6-figure supplement 4, Figure 7, Figure 8, and Figure 9 and Figure 10.
